# P-202. 7- Year experience with Clostridioides difficile Infection (CDI): pre- and post-COVID-19 pandemic, effect of diagnostic stewardship and the gender difference

**DOI:** 10.1093/ofid/ofae631.406

**Published:** 2025-01-29

**Authors:** Mariam Younas, Ann Newell, Anthony Armor, Carlos Rios-Bedoya

**Affiliations:** Hurley Medical Center/Michigan State University College of Human Medicine, Flint, Michigan; Hurley Medical Center, Flint, Michigan; Hurley Medical Center, Flint, Michigan; Hurley Medical Center, Flint, Michigan

## Abstract

**Background:**

Diagnostic stewardship interventions, such as order sets, reflex orders, hard and soft stop alerts and decision algorithms have shown to improve facility C. difficile rates. We aimed to examine the impact of COVID-19 infection and diagnostic stewardship on incidence and prevalence of CDI at our institution, based on gender.

**Figure:**
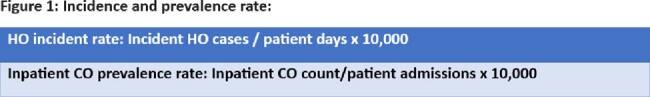

**Methods:**

Using National Healthcare Safety Network (NHSN) surveillance Laboratory Identification (Lab ID) events, 926 CDI cases, ≥ 18 years of age were identified among patients hospitalized at Hurley Medical Center from 01/2015 to 07/2022. NHSN surveillance definitions were used to describe the incident, recurrent cases and determine the epidemiologic categories. Hospital onset (HO) incident rate is described as per 10,000 patient days and inpatient community-onset (CO) CDI prevalence rate described as per 10,000 admissions (Figure 1).

**Table 1:**
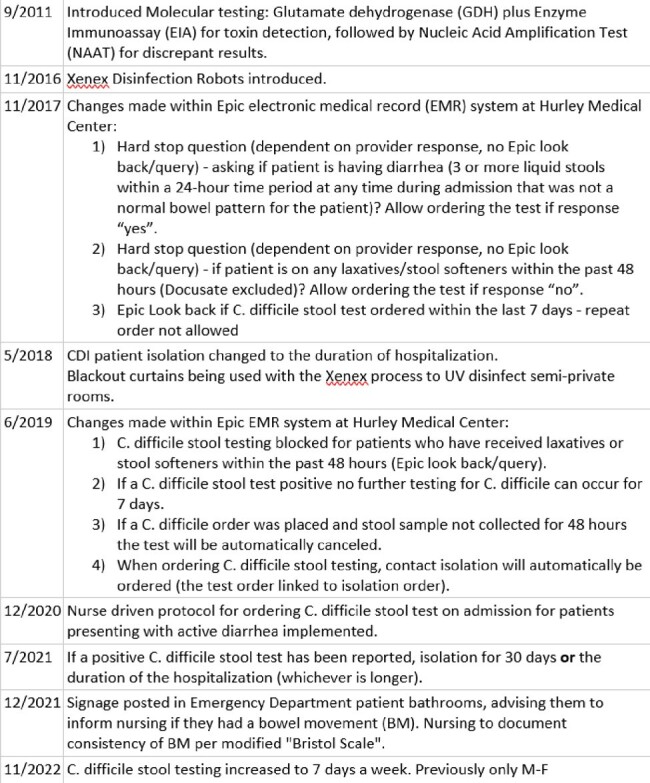
Diagnostic stewardship and infection control interventions

**Results:**

During the 7.6-year study period, overall HO CDI incident rate and inpatient CO CDI prevalence rate decreased, a percentage decrease of 34% and 53% respectively. The rates were lowest in 2020, onset of COVID-19 pandemic (percentage decrease of 22% for HO CDI incident and 7% for CO CDI prevalence rates as compared to 2019 pre-COVID-19 pandemic). There was a transient increase in 2021 (percentage increase of 36% for HO CDI incident and 14% for CO CDI prevalence rates as compared to 2019) (Figure 2, 3). HO CDI incident rates were slightly higher in men (average 11.4% difference) as compared to women except in 2017. CO CDI prevalence rates were much higher in women as compared to the men (average 13.4% difference) with the exception of 2017 and 2022.

**Figure 2:**
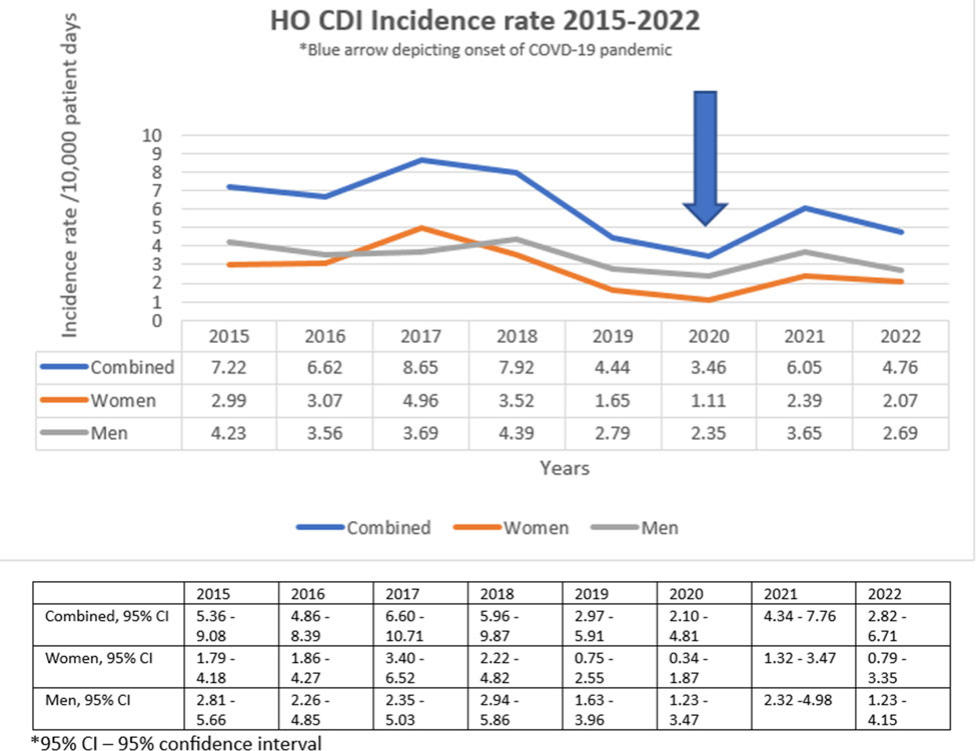
HO CDI Incidence rate 2015-2022, with 95% confidence intervals

**Conclusion:**

HO CDI incident rate and inpatient CO CDI prevalence rates were lowest in 2020 (the onset of COVID-19 pandemic), likely secondary to computerized clinical decision support (CCDS) interventions introduced in 2019 (Table 1). The transient increase in rates seen in 2021, was likely due to increase in antibiotic utilization during the pandemic. Diagnostic stewardship played a key role in optimizing the diagnosis of CDI and improving CDI rates at our institution. There was a discrepancy of the CO CDI and HO CDI rates between the sexes, likely owing to the higher outpatient antibiotic use in women as compared to men, highlighting the importance of ambulatory stewardship.

**Figure 3:**
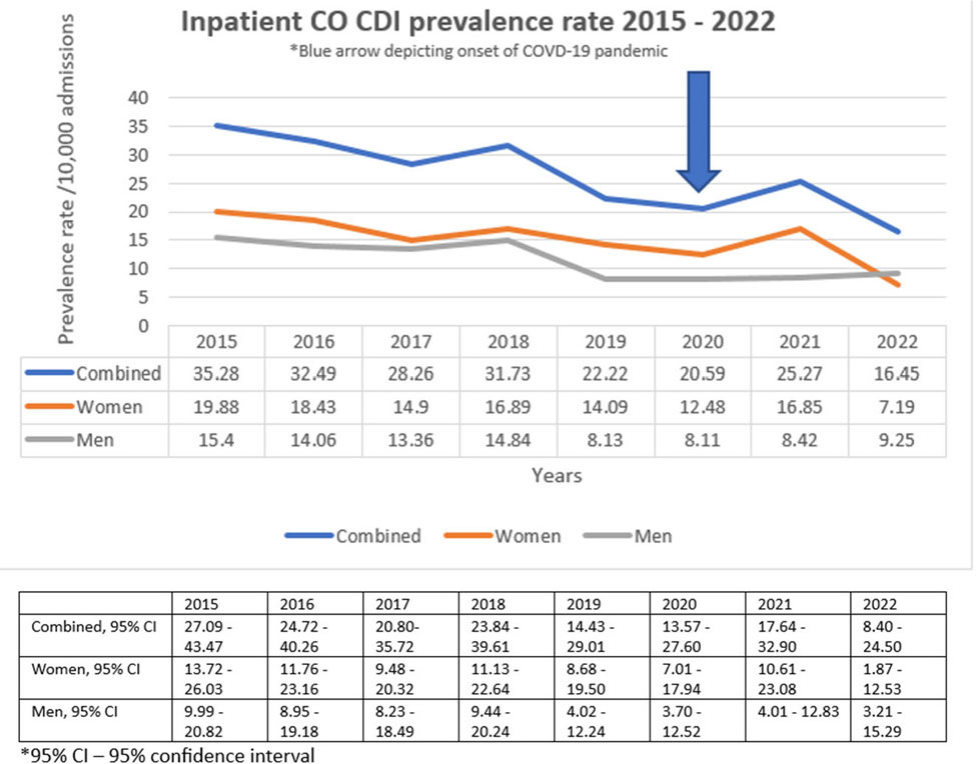
Inpatient CO CDI prevalence rate, with 95% confidence intervals

**Disclosures:**

**All Authors**: No reported disclosures

